# Clinical implications and prognostic value of mastoid effusion in the management of aneurysmal subarachnoid hemorrhage

**DOI:** 10.3389/fneur.2025.1603869

**Published:** 2025-08-06

**Authors:** Junhyung Kim, Sohyun Kim, Chang Ki Jang, Hyun Jin Han, Keun Young Park, Jung-Jae Kim, Yong Bae Kim, Jiwoong Oh

**Affiliations:** ^1^Department of Neurosurgery, Yonsei University College of Medicine, Seoul, Republic of Korea; ^2^Department of Physiology, Yonsei University College of Medicine, Seoul, Republic of Korea

**Keywords:** mastoid effusion, middle ear effusion, intracranial pressure, subarachnoid hemorrhage, aneurysm, vasospasm

## Abstract

**Background:**

The clinical significance of mastoid effusion (ME) in intensive care unit (ICU) patients has not been well elucidated. Recently, an association between ME and intracranial pressure (ICP) has been reported. We aimed to investigate the clinical implications of ME occurrence in the management of aneurysmal subarachnoid hemorrhage (aSAH) patients and its association with their prognosis.

**Methods:**

Data from patients aged > 18 years who were treated for aSAH in a single institution between January 2020 and December 2022 were retrospectively reviewed. Brain CT or MRI images obtained within the first 14 days after the onset of SAH were evaluated for the presence of ME, which is defined as either opacification or an air-fluid level in the mastoid air cells. We examined the patients’ demographic information, neurological and medical status at admission, aneurysm and treatment characteristics, and clinical outcomes. We then analyzed how these factors were associated with the occurrence of ME.

**Results:**

A total of 114 patients were included in the study. ME was observed in 40 patients (34.5%) within the first 14 days, occurring at a mean of 5.0 ± 3.5 days after the onset of SAH. In multivariate analysis, patients with ME were found to have a higher incidence of tracheostomy (odds ratio [OR] 10.034, *p* = 0.024), radiologic vasospasm (OR 4.987, *p* = 0.018), a higher APACHE II score (OR 1.138, *p* = 0.013), and poor clinical outcomes (OR 4.289, *p* = 0.041), defined as modified Rankin Scale score > 2 at 90 days. Poor clinical outcomes were independently associated with ME (OR 5.003, *p* = 0.006).

**Conclusion:**

This study demonstrated that ME was observed in 34.5% of aSAH patients and was associated with poor clinical outcomes. ME may serve as a simple and useful prognostic indicator for predicting poor outcomes in aSAH patients.

## Introduction

Mastoid effusion (ME) occurs frequently along with middle ear effusion in children as a consequence of the accumulation of transudate caused by negative pressure or inflammation in the middle ear (1). Due to the differences in the orientation, length, and function of the Eustachian tube, ME is not a common finding in healthy adults, with an incidence of approximately 1% (2, 3). However, several studies have demonstrated that the incidence of ME is higher in intensive care unit (ICU) patients than in general population. Risk factors for the development of ME in ICU patients include old age, increased mucus secretion caused by prolonged endotracheal intubation for mechanical ventilation and a nasogastric tube, prolonged ICU stay, and altered mental status, many of which can be attributed to the anatomical connection of the middle ear space and the nasopharynx via the Eustachian tube (2, 4, 5).

Anatomical continuity also exists between the subarachnoid space and the cochlear aqueduct of the middle ear, and it provides the background for the development of a non-invasive tool for monitoring intracranial pressure (ICP) by measuring the tympanic membrane pressure (6, 7). Moreover, a recent study reported that elevated levels of ICP was associated with the occurrence of ME in neuro-ICU patients (8). Therefore, it can be postulated that the occurrence of ME in patients with neurologic insults who are at a risk of ICP variation may offer additional information regarding the clinical course and outcomes.

Subarachnoid hemorrhage (SAH) due to intracranial aneurysm rupture is a disastrous event with high morbidity and mortality rates (9, 10). A rupture of an aneurysm causes an abrupt and delayed increase in ICP, resulting in impaired cerebral perfusion and temporary intracranial circulatory arrest (11). Increased ICP plays an important role in the clinical course of aneurysmal subarachnoid hemorrhage (aSAH) patients, as it is related to common complications of aSAH such as acute hydrocephalus or delayed cerebral ischemia, which mostly occur in the first 14 days after the ictus. We hypothesized that patients receiving aSAH management who developed ME in the first 14 days would demonstrate unfavorable clinical outcomes. The purpose of this study was to investigate the incidence and the clinical significance of ME in patients with aSAH.

## Methods

This retrospective study was approved by our Institutional Review Board and was performed under the guidelines outlined in the Declaration of Helsinki. The diagnosis of ME was based on the criteria proposed by J. Gossner, and only cases classified as “marked” (i.e., fluid signal involving more than half of the mastoid air cells) were considered ME-positive. By including only clearly defined cases, we minimized subjectivity in the interpretation. Accordingly, there was no interobserver disagreement between the neurovascular specialist and the neuro-intensivist who independently reviewed the images. This study follows the STROBE guidelines for retrospective studies.

### Study population

Patient data between January 2020 and December 2022 were obtained from our institution’s prospectively maintained aSAH database. Adult patients over the age of 18 who were admitted to the neuro-ICU of our institution for the treatment and management of aSAH were included. The exclusion criteria were as follows: (1) incomplete medical records, (2) transfer to our institution more than 1 day after the onset of aSAH, and (3) absence of follow-up brain computed tomography (CT) or magnetic resonance imaging (MRI) within 14 days to assess for ME.

### Management of aSAH patients

We adhered to the standard treatment strategy for aSAH. All patients who presented with acute-phase aSAH were admitted to the neuro-ICU and received cerebral angiography within 24 h, unless contraindicated. Except for those who did not wish to receive surgical or endovascular treatment, patients received microsurgery or endovascular treatment to secure the ruptured aneurysm. To manage increased ICP, external ventricular drainage, lumbar drainage, or decompressive surgery was performed. Intravenous or oral nimodipine was administered to prevent post-SAH vasospasm. Brain CT, brain MRI, or magnetic resonance angiography (MRA) were conducted when necessary. Patients with a good initial Hunt–Hess grade who were expected to be at a low risk for post-SAH complications were transferred to the general ward as early as 3 to 7 days after ictus, but those at moderate to high risk were monitored in the neuro-ICU for at least 10 to 14 days.

### Radiological assessment for ME and vasospasm

Non-contrast brain CT or brain CT angiography was the primary modality for radiologic evaluation of aSAH patients, with brain MRI or MRA performed as needed. The presence of ME was defined as partial or complete opacification of the mastoid air cell cavity, showing an air-fluid level in non-contrast brain CT, or high signal intensity in T2-weighted MR images on either or both sides. The images obtained within the first 14 days after the onset of SAH were independently reviewed by a neurovascular specialist and a neuro-intensivist. They assessed the presence of ME and reached consensus through discussion.

Radiologic vasospasm was evaluated using a standardized institutional protocol. Daily transcranial Doppler (TCD) monitoring was performed in all patients. If TCD findings were suggestive of vasospasm, CTA was immediately performed to confirm the diagnosis. In patients without vasospasm findings on TCD, routine CTA was performed approximately 1 to 2 weeks after the day of rupture to evaluate vascular status. Vasospasm was ultimately diagnosed when luminal narrowing of ≥30% was observed on CTA compared to baseline vascular imaging.

### Clinical assessment

At admission, the Glasgow Coma Scale (GCS) and Hunt–Hess grade, as well as the Acute Physiology and Chronic Health Evaluation II (APACHE II) scores, were used to assess the initial neurological condition. The duration of neuro-ICU stay and mechanical ventilation, ventriculoperitoneal shunt within 90 days, and modified Rankin Scale (mRS) score at 90 days were recorded. We defined poor clinical outcome as 90-day mRS > 2. Symptomatic vasospasm was diagnosed when vasospasm was radiologically confirmed and patients revealed neurological worsening with no other identifiable causes (12).

### Statistical analysis

Statistical analysis was performed using SPSS Statistics 25.0 (IBM). Fisher’s exact test or χ (2) test was performed for categorical variables. Mann–Whitney U-test or Student’s *t*-test was performed for continuous variables of clinical outcomes and the univariate analysis of the factors associated with ME and poor clinical outcomes. All variables with clinical importance were introduced into a multivariate analysis using the binary logistic regression method. A *p-*value of < 0.05 was considered statistically significant.

## Results

### Patient and aneurysm characteristics

During the study period, 121 patients with ruptured intracerebral aneurysms were treated in our institution. Excluding one patient who was transferred to our institution a few days after the onset of aSAH and six patients who had not taken any follow-up brain CT or MRI images, a total of 114 patients (mean age, 59.5 ± 14.2 years; male/female ratio = 35:79) treated for aSAH were included for analysis. A total of 62 patients (54.4%) initially presented with a Hunt–Hess grade greater than 2 at admission. The majority of the aneurysms were saccular (*n* = 110, 96.5%) in the anterior circulation (*n* = 95, 83.3%). These characteristics are summarized in Table 1.

**Table 1:** Patient and aneurysm characteristics.

Variables	Values
Patients	114
Age, mean	59.5 ± 14.2
Male sex, *n* (%)	35 (31.7)
Comorbidities, *n* (%)	
Hypertension	47 (41.2)
Diabetes mellitus	9 (7.9)
Dyslipidemia	18 (15.8)
Smoking	23 (20.2)
Aneurysm location, *n* (%)	
Anterior circulation	95 (83.3)
Posterior circulation	19 (16.7)
Aneurysm type, *n* (%)	
Saccular	110 (96.5)
Mycotic	1 (0.9)
Dissecting	3 (2.6)
Initial Hunt–Hess Grade, *n* (%)	
1	0 (0)
2	52 (45.6)
3	34 (29.8)
4	24 (21.1)
5	4 (3.5)
Initial GCS, median	14 (11–15)
APACHE II score, mean	17.2 ± 7.9

Values are presented in mean ± standard deviation, median (interquartile range), or number (%).

APACHE II, Initial Acute Physiology and Chronic Health Evaluation II; GCS, Glasgow Coma Scale.

### Treatment characteristics and outcomes

Endovascular treatment was the dominant modality (81.6%) for securing ruptured aneurysms. A total of 24 patients (21.1%) received ventriculoperitoneal shunt operation within 90 days, and 16 patients (14.0%) received tracheostomy. Patients were treated in the neuro-ICU for a median duration of 13 days (interquartile range [IQR] 8–19 days). ME was observed in 40 patients (34.5%) at a mean of 5.0 ± 3.5 days after the onset of aSAH. Poor clinical outcomes were demonstrated by 41 patients (36.0%). These characteristics are summarized in Table 2.

**Table 2:** Treatment characteristics and outcomes.

Variables	Values
Treatment modality, *n* (%)	
Microsurgery	21 (18.4)
Endovascular treatment	93 (81.6)
Extraventricular drainage, *n* (%)	36 (31.6)
Ventriculoperitoneal shunt, *n* (%)	24 (21.1)
Tracheostomy, *n* (%)	16 (14.0)
Neuro-ICU stay duration, median, days	13 (8–19)
ME within first 14 days (%)	40 (34.5)
Interval between aSAH and ME, mean, days	5.0 ± 3.5
Poor clinical outcome*, *n* (%)	41 (36)

Values are presented in mean ± standard deviation, median (interquartile range), or number (%).

aSAH; aneurysmal subarachnoid hemorrhage; ICU, intensive care unit; ME, mastoid effusion.

^*^Poor clinical outcome was defined as a 90-day modified Rankin Scale > 2.

### Factors associated with the occurrence of ME

**Figure 1:**
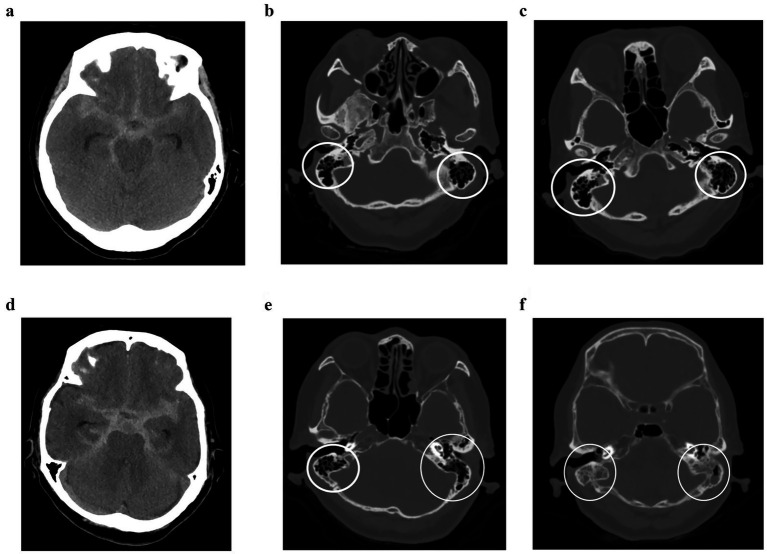
Mastoid effusion (ME) in patients with subarachnoid hemorrhage (SAH). **(a–c)** Case with absence of ME. A 66-year-old woman with an initial Fisher’s grade III SAH. The initial brain CT scan showed no evidence of ME. A follow-up CT scan 2 week later also showed no signs of ME (white circle). The patient’s modified Rankin Scale (mRS) score at post-operative 90 days was 0. **(d–f)** Case with the presence of ME. An 80-year-old woman with Fisher’s grade III SAH on the initial CT scan, with no signs of ME. Two weeks later, a follow-up CT scan revealed bilateral ME (white circle). The patient had an mRS score of 4 at post-operative 90 days.

In univariate analysis, the occurrence of ME was statistically associated with older age (*p* = 0.002), male sex (*p* = 0.034), and ruptured aneurysms in the posterior circulation (*p* = 0.005). Patients with ME had a higher likelihood of presenting with an initial Hunt–Hess grade > 2 (80.0% vs. 40.5%, *p* < 0.001), higher APACHE II scores (22.2 ± 8.3 vs. 14.5 ± 6.2, *p* < 0.001), and longer durations of neuro-ICU stay (19 days vs. 10 days, *p* < 0.001) and mechanical ventilation (13 days vs. 0 days, *p* < 0.001) compared to those without ME. Furthermore, the rates of radiologic vasospasm and symptomatic vasospasm were significantly higher in patients with ME compared to those without ME (50.0% vs. 18.5%, *p* = 0.002 for radiologic vasospasm; 30.0% vs. 7.7%, *p* = 0.01 for symptomatic vasospasm). Terson’s syndrome was diagnosed in two patients, both of whom developed ME. ME was associated with poor clinical outcomes at 90 days (*p* < 0.001). There was no significant relationship between the occurrence of ME and the types of treatment modality for securing the aneurysms. In multivariate analysis, tracheostomy (odds ratio [OR] 10.034, *p* = 0.024), radiologic vasospasm (OR 4.987, *p* = 0.018), a higher APACHE II score (OR 1.138, *p* = 0.013), and a poor clinical outcome (OR 4.289, *p* = 0.041) were independently associated with ME occurrence. These results are summarized in Table 3 and Figure 1.

**Table 3:** Univariate and multivariate analyses for the risk factors of ME.

Variables	Univariate analysis			Multivariate analysis	
	No effusion (*n* = 74)	Effusion (*n* = 40)	*p*-value	OR (95% CI)	*p*-value
Age	56.5 ± 14.4	65.0 ± 12.2	0.002		
Male sex	18 (23.3)	17 (43.9)	0.034		
Comorbidities					
Hypertension	27 (36.5)	20 (50.0)	0.162		
Diabetes mellitus	6 (8.1)	3 (7.5)	>0.999		
Dyslipidemia	11 (14.9)	7 (17.5)	0.713		
Smoking	17 (23.0)	6 (15.0)	0.311		
Aneurysm in posterior circulation	7 (9.5)	12 (30.0)	0.005		
Initial Hunt–Hess Grade			<0.001		
≤ 2	44 (59.5)	8 (20.0)			
> 2	30 (40.5)	32 (80.0)			
Initial Intracranial pressure (mmHg)	20.9 ± 11.4 (*n* = 20)	19.2 ± 9.6 (*n* = 28)	0.107		
Initial GCS	15 (13–15)	8 (5–13)	<0.001		
Initial APACHE II	14.5 ± 6.2	22.2 ± 8.3	<0.001	1.138 (1.027–1.261)	0.013
Initial WBC	10,068 (3703)	11,888 (4439)	0.021		
Initial CRP†	3.5 (0.9–9.0)	3.3 (1.08–12.38)	0.787		
Initial DNI††	1.44 (2.96)	2.27 (3.23)	0.166		
Treatment modality for aneurysm			0.749		
Microsurgery	13 (17.6)	8 (20.0)			
Endovascular treatment	61 (82.4)	33 (82.5)	>0.999		
External ventricular drainage	13 (17.6)	23 (57.5)	<0.001		
Tracheostomy	2 (2.7)	14 (35.0)	<0.001	10.034 (1.352–74.448)	0.024
Ventriculoperitoneal shunt	9 (12.2)	15 (37.5)	0.002		
Hydrocephalus	15 (20.3)	27 (67.5)	<0.001		
Neuro-ICU stay duration (day)	10 (6–13)	19 (14–26)	<0.001		
Ventilator maintenance duration	0 (0–1)	13 (6–20)	<0.001		
Radiologic vasospasm*	12 (18.5)	15 (50.0)	0.002	4.987 (1.322–18.814)	0.018
Symptomatic vasospasm*	5 (7.7)	9 (30.0)	0.01		
Terson’s syndrome	0 (0)	2 (5.0)	0.127		
Poor clinical outcome	12 (16.2)	29 (72.5)	<0.001	4.289 (1.061–17.333)	0.041

Values are presented as mean ± standard deviation, median (interquartile range), or number (%).

APACHE II, Initial Acute Physiology and Chronic Health Evaluation II; CI, confidence interval; CRP, C-reactive protein; DNI, delta neutrophil index; GCS, Glasgow Coma Scale; ICU, intensive care unit; OR, odds ratio; WBC, white blood cell.

† This variable was evaluated in 109 patients.

†† This variable was evaluated in 98 patients.

^*^This variable was evaluated in 95 patients.

### Factors associated with poor clinical outcomes

Univariate analysis revealed that a poor clinical outcome was associated with several factors that have been known as risk factors for aSAH, such as older age (*p* < 0.001), posterior circulation aneurysm (*p* = 0.007), and a higher Hunt–Hess grade (*p* < 0.001). Compared to patients who had good clinical outcomes, those with poor clinical outcomes had prolonged neuro-ICU care (10 vs. 19 days, *p* < 0.001) and mechanical ventilation (0 vs. 12 days, *p* < 0.001) and experienced a higher occurrence of ME (15.1% vs. 70.7%, *p* < 0.001). In multivariate analysis, along with older age (OR 1.081, *p* = 0.001) and Hunt–Hess grade > 2 (OR 4.515, *p* = 0.014), ME occurrence (OR 5.003, *p* = 0.006) was statistically associated with a poor clinical outcome. These results are presented in Table 4.

**Table 4:** Univariate and multivariate analyses for risk factors of poor clinical outcome in aSAH patients.

Variables	Univariate analysis			Multivariate analysis	
	Good outcome (*n* = 73)	Poor outcome (*n* = 41)	*p*-value	OR (95% CI)	*p*-value
Age	55.0 ± 13.6	67.5 ± 11.4	<0.001	1.081 (1.032–1.132)	0.001
Male sex	22 (31.1)	13 (31.7)	>0.999		
Comorbidities			0.406		
Hypertension	29 (38.4)	19 (46.3)			
Diabetes mellitus	4 (5.5)	5 (12.2)	0.279		
Dyslipidemia	8 (11.0)	10 (24.4)	0.059		
Smoking	19 (26.0)	4 (9.8)	0.038		
Aneurysm in the posterior circulation	7 (9.6)	12 (29.3)	0.007		
Initial Hunt–Hess Grade			<0.001		
≤ 2	44 (60.3)	8 (19.5)			
> 2	29 (39.7)	33 (80.5)		4.515 (1.357–15.030)	0.014
Initial GCS	15 (13–15)	9 (5–13.5)	<0.001		
Initial APACHE II	14.3 ± 6.3	22.5 ± 7.8	<0.001		
Initial WBC	10,616 ± 3,823	10,914 ± 4,494	0.839		
Initial CRP†	3.5 (1.05–9.35)	3.5 (1.03–9.00)	0.905		
Initial DNI††	1.44 ± 2.81	2.29 ± 3.47	0.152		
Treatment modality for aneurysm			0.822		
Microsurgery	13 (17.8)	8 (19.5)			
Endovascular treatment	60 (82.2)	34 (82.9)			
External ventricular drainage	15 (20.5)	21 (51.2)	0.001		
Tracheostomy	3 (4.1)	13 (31.7)	<0.001		
Ventriculoperitoneal shunt	10 (13.7)	14 (34.1)	0.01		
Neuro-ICU stay duration	10 (5–13)	19 (13–26)	<0.001		
Ventilator maintenance duration	0 (0–1)	12 (2–19)	<0.001		
Radiologic vasospasm*	16 (25.0)	11 (35.5)	0.288		
Symptomatic vasospasm*	7 (10.9)	7 (22.6)	0.215		
ME	11 (15.1)	29 (70.7)	<0.001	5.003 (1.570–15.935)	0.006

Values are presented as mean ± standard deviation, median (interquartile range), or number (%).

aSAH, aneurysmal subarachnoid hemorrhage; APACHE II, Initial Acute Physiology and Chronic Health Evaluation II; CI, confidence interval; CRP, C-reactive protein; DNI, delta neutrophil index; GCS, Glasgow Coma Scale; ICU, intensive care unit; ME, mastoid effusion; OR, odds ratio; WBC, white blood cell.

† This variable was evaluated in 109 patients.

†† This variable was evaluated in 98 patients.

^*^This variable was evaluated in 95 patients.

### Subgroup analysis by neurological grade and treatment modality

To further explore the relationship between mastoid effusion (ME) and neurological severity at admission, a subgroup analysis was conducted based on the Hunt–Hess (HH) grade. Patients were categorized into two groups: HH ≤ 2 and HH > 2. The occurrence of ME was significantly higher in the HH > 2 group (51.6%) compared to the HH ≤ 2 group (15.4%) (*p* < 0.001), suggesting a strong association between a poor neurological grade and the presence of ME.

In addition, a subgroup analysis was performed among 94 patients who underwent endovascular treatment. In this subgroup, multivariate logistic regression revealed that the presence of ME was an independent predictor of poor clinical outcomes (OR, 4.079; 95% CI, 1.060–15.691; *p* = 0.041), even after adjusting for age, initial Glasgow Coma Scale (GCS) score, and HH grade (Table 5). These findings support the prognostic significance of ME in patients with aSAH, particularly those treated with endovascular intervention.

**Table 5:** Univariate and multivariate analyses of risk factors for poor clinical outcome in aSAH patients treated with endovascular treatment.

Variables	Univariate analysis			Multivariate analysis	
	Good outcome (*n* = 60)	Poor outcome (*n* = 34)	*p*-value	OR (95% CI)	*p*-value
Age	55.0 ± 14.1	67.6 ± 11.5	<0.001	1.093 (1.030–1.159)	0.003
Male sex	19 (31.7)	11 (32.4)	0.945		
Comorbidities					
Hypertension	25 (41.7)	17 (50.0)	0.435		
Diabetes mellitus	4 (6.7)	5 (14.7)	0.277		
Dyslipidemia	8 (13.3)	10 (29.4)	0.057		
Smoking	14 (23.3)	3 (8.8)	0.079		
Aneurysm in the posterior circulation	7 (11.7)	12 (35.3)	0.006		
Initial Hunt–Hess Grade > 2	24 (40.0)	29 (85.3)	<0.001	4.686 (1.032–21.279)	0.045
Initial GCS	15 (13–15)	8 (4.25–13)	<0.001	4.515 (1.357–15.030)	0.014
Initial APACHE II	14.8 ± 6.3	23.6 ± 7.9	<0.001		
Initial WBC	10,423 ± 3,766	11,176 ± 4,385	0.382		
Initial CRP	3.8 (1.1–11.7)	3.5 (1.0–9.0)	0.662		
Initial DNI	1.46 ± 2.88	1.90 ± 3.14	0.496		
External ventricular drainage	10 (16.7)	18 (52.9)	0.001		
Tracheostomy	2 (3.3)	11 (32.4)	<0.001		
Ventriculoperitoneal shunt	9 (15.0)	13 (38.2)	0.011		
Neuro-ICU stay duration	10 (5–13)	20.5 (13.25–29)	<0.001		
Ventilator maintenance duration	0 (0–1)	12.5 (3–19.75)	<0.001		
Radiologic vasospasm*	11 (20.4)	8 (32.0)	0.261		
Symptomatic vasospasm*	4 (7.4)	4 (16.0)	0.254		
ME	9 (15.0)	24 (70.6)		4.079 (1.060–15.691)	0.041

Values are presented as mean ± standard deviation, median (interquartile range), or number (%).

APACHE II, Initial Acute Physiology and Chronic Health Evaluation II; CI, confidence interval; CRP, C-reactive protein; DNI, delta neutrophil index; GCS, Glasgow Coma Scale; ICU, intensive care unit; ME, mastoid effusion; OR, odds ratio; WBC, white blood cell. *This variable was evaluated in 79 patients.

## Discussion

In this retrospective study, we found that ME occurred in 34.5% of aSAH patients within the first 14 days (mean 5.0 ± 3.5 days) and was associated with radiologic vasospasm and poor clinical outcomes. Moreover, along with older age and a poor Hunt–Hess grade, ME was an independent risk factor for poor clinical outcomes in aSAH patients.

Several studies have reported a higher incidence of ME in ICU patients than in the healthy adult population, ranging between 10.3 and 53% (2, 8, 13). Risk factors for the development of ME include thickened oropharyngeal secretions due to mucosal irritation by endotracheal intubation and nasogastric tube, and decreased mentality impairing the patient’s ability to clear excessive mucosal secretion and to open the Eustachian tube, which are hardly modifiable (2, 4, 13). Although ME can potentially cause acute mastoiditis and develop into intracranial complications such as meningitis, empyema, and brain abscess (14), incidentally detected MEs are rarely related to temporal bone disease (15). ME observed in ICU patients is considered benign in most cases, obscuring its clinical significance (2, 13). This study, however, showed that ME was independently associated with poor clinical outcomes in aSAH patients. Patients who suffer aSAH are usually hospitalized for 14 to 21 days due to the possible occurrence of delayed complications (16), allowing prognostication to be made at least 14 days after the ictus in most cases. In conjunction with the established risk factors for poor outcomes in aSAH patients, such as Hunt–Hess score, increasing age, and ruptured posterior circulation aneurysm, the occurrence of ME may offer an additional prognostic value.

Recently, Jung et al. suggested that increased ICP was associated with ME occurrence in patients who underwent intracranial surgery (8). The authors revealed that the prediction model for the development of ME improved when peak ICP values were included in the model. Timely recognition and proper management of increased ICP are critical for improving clinical outcomes in patients with aSAH. However, most widely used ICP monitoring tools, such as external ventricular drainage, are invasive, and it is often a dilemma whether to place invasive ICP monitoring devices due to the risks of infection or hemorrhage (17, 18). It has been recognized that ICP is transmitted to the perilymphatic space in the middle ear, and the middle ear pressure represented by tympanic membrane displacement can be utilized for the indirect measurement of ICP (6, 19). Assuming that the occurrence of ME reflects increased ICP, a clinician may conduct additional evaluations or procedures to assess the patient’s ICP status, or ophthalmologic evaluation for the possibility of Terson’s syndrome when ME is observed. Although this finding was not statistically significant, Terson’s syndrome, a consequence of elevated ICP, was diagnosed in two patients who developed ME in this study. However, whether there is a causal relationship between elevated ICP and the development of ME is not known. A few studies attempted to explain the development of ME in patients with lateral sinus thrombosis as the result of venous congestion rather than the Eustachian tube dysfunction, as the laterality of the ME coincided with the intracranial lesions responsible for the elevated ICP (8, 20, 21). Such an explanation may not fit in other types of intracranial pathologies not involving venous congestion. From the anatomical perspective, the movement of cerebrospinal fluid from the subarachnoid space to the mastoid air cells via the middle ear due to a pressure gradient is theoretically possible. The relationship between elevated ICP and the development of ME, their mechanisms, and temporal associations should be further investigated to refine the clinical significance of ME in patients whose ICP assessments are critical.

Another finding in this study was that the occurrence of ME was associated with post-SAH vasospasm. Post-SAH vasospasm has multiple risk factors, including the amount of SAH, presence of intracranial hemorrhage and intraventricular hemorrhage, female sex, and increased ICP (22). Fukuhara et al. demonstrated that elevated ICP was associated with the development and the duration of vasospasm after aSAH (23). Similarly, Gambardella et al. showed that the use of osmotic diuretics to control ICP lowered the risk of developing delayed cerebral ischemia in patients with aSAH (24). On the other hand, Heuer et al. reported only a weak relationship between ICP and the development of angiographic or symptomatic vasospasm (25). The authors provided a few explanations for the weak link. They argue that patients with severely elevated ICP died before vasospasm could occur, or did not undergo follow-up angiographic evaluations. In our case, 8 patients (42.1%) out of 19 patients did not undergo follow-up angiography studies, as poor prognosis was expected.

The mean interval between the onset of aSAH and detection of ME was 5 days. Limited studies have reported the time for ME to develop in ICU patients. Jung et al. reported a mean of 11.1 days for the development of ME in patients who underwent intracranial surgery, and Huyett et al. described that ME was a late finding and prevalent in patients with a prolonged ICU stay (2, 8). Considering that elevated ICP contributes to the development of ME, it can be postulated that ME in patients with neurological insults such as SAH would develop at an earlier period than in patients with other etiologies. Early brain injury after aSAH is known as a pathologic process that occurs in the first 72 h, and involves the elevation of ICP, reduction of cerebral blood flow, and neuronal cell death (26). SAH-induced vasospasms usually occur 4 to 14 days after the ictus, with a peak incidence at 7 days (27). The relationship between the early phase of brain injury and the development of ME should be further described in future studies.

Although our findings suggest that mastoid effusion (ME) is associated with poor outcomes and angiographic vasospasm in patients with aSAH, the exact pathophysiological mechanisms underlying ME development in neurologically injured patients remain uncertain. It has been hypothesized that elevated intracranial pressure (ICP) or impaired cerebrospinal fluid drainage may contribute to fluid accumulation within mastoid air cells. However, this mechanism has not yet been established in the existing literature.

Given the routine use of non-contrast brain CT in the acute-phase SAH, ME may serve as a readily identifiable imaging marker that reflects disease severity or elevated ICP, particularly in patients without invasive monitoring. While ME is not a direct therapeutic target, its presence may have potential implications for early risk stratification and the intensity of subsequent monitoring or intervention. Nonetheless, this study was not designed to establish a causal mechanism or to propose a clinical algorithm based on ME findings. Further prospective studies are needed to clarify the biological basis of ME in this patient population and to determine how it may be effectively incorporated into clinical decision-making processes. We acknowledge this limitation and have highlighted the need for future research in this area.

Previous studies have suggested a potential correlation between mastoid effusion (ME) and elevated intracranial pressure (ICP), implying that ME may serve as an indirect radiological marker of increased ICP in patients with acute brain injury (8). One proposed mechanism is that a pressure gradient between the subarachnoid space and the middle ear cavity could facilitate the movement of cerebrospinal fluid (CSF) into the mastoid air cells, resulting in effusion. Furthermore, elevated ICP has been linked to both the occurrence and persistence of cerebral vasospasm following aSAH, potentially explaining the association between ME and poor outcomes observed in this study.

To further explore this hypothesis, we analyzed available ICP data in relation to ME status (Table 3). The mean ICP was 20.9 ± 11.4 mmHg in the ME-negative group and 19.2 ± 9.6 mmHg in the ME-positive group, with no statistically significant difference. This finding should be interpreted cautiously for several reasons. First, the timing of ICP measurement and the detection of ME on imaging did not always coincide, limiting a temporal correlation. Second, most patients underwent intensive ICP-lowering interventions, including osmotherapy, targeted temperature management, and coma therapy, which may have modified or masked actual ICP values. These factors likely contributed to the lack of observed statistical significance.

Nevertheless, we believe that ME may still reflect transient or unrecorded ICP elevations, particularly in patients without invasive monitoring. Given that non-contrast brain CT is routinely performed in the acute phase of aSAH, the detection of ME may provide a non-invasive and easily accessible clue suggesting raised ICP, especially in resource-limited settings.

Importantly, the pathophysiology of ICP elevation in aSAH differs from that of other neurological conditions such as traumatic brain injury or ischemic stroke. In aSAH, abrupt ICP elevation can occur due to aneurysmal rupture and subsequent hydrocephalus and can fluctuate rapidly with events such as rebleeding, vasospasm, or therapeutic interventions. These unique dynamics complicate the interpretation of single-timepoint ICP data but underscore the potential value of adjunctive imaging markers such as ME.

This study has several limitations. Due to its retrospective design and small sample size, selection and information bias were unavoidable, as the details of management and follow-up strategies for aSAH patients varied. For example, the follow-up non-contrast brain CT imaging intervals varied between patients, obscuring the precise timing of ME development, and only 83.3% (95/114) of the patients underwent follow-up angiography studies at irregular intervals to be evaluated for the occurrence of vasospasm. In asymptomatic patients with longstanding mastoid effusion resulting from conditions such as chronic otitis media, the absence of prior imaging may lead to misclassification of ME as a newly developed finding. While ME could be ruled out through comparison with previous imaging in patients who underwent serial follow-up studies, this was not feasible in newly admitted patients without prior imaging. The likelihood of pre-existing mastoiditis was presumed to be comparable between the two groups. Furthermore, as mastoid effusion grade 2 or higher was defined as ME-positive in this study, cases of mild mastoiditis were likely excluded. Patients with a documented history of otitis media were also excluded based on a thorough review of medical records. In our dataset, only 5 out of the 40 patients with ME showed evidence of ME on their initial brain CT. Although it was not possible to determine whether these cases represented pre-existing ME, all five patients demonstrated progression of ME on follow-up imaging.

Another limitation is the potential subjectivity in the radiologic diagnosis of ME. To address this limitation, only clearly defined cases meeting the “marked” criteria proposed by Gossner—defined as a fluid signal involving more than half of the mastoid air cell cavity—were included (28). All imaging was independently assessed by a neurovascular specialist and a neuro-intensivist, and there was complete agreement between the two evaluators. Therefore, a formal consistency test was not conducted. Additionally, although a quantitative threshold such as the Hounsfield unit (HU) could be useful, variability in CT protocols and scanner calibration limited its application in this retrospective analysis. Finally, some factors that may influence the development of ME—such as the presence of nasogastric tubes or ICP values—were not included in this study. In particular, the absence of ICP data may limit the ability to fully explore the relationship between ME and elevated intracranial pressure. Despite these limitations, ME was found to be independently associated with poor outcomes and cerebral vasospasm in patients with aSAH. Further studies are warranted to clarify the underlying mechanisms linking ME with elevated levels of ICP and to validate its role as a prognostic marker.

## Conclusion

This study demonstrated that the occurrence of ME in aSAH patients was independently associated with tracheostomy, vasospasm, and poor clinical outcomes. Given that ME can be easily identified on non-contrast brain CT early in the clinical course, it may serve as an additional imaging marker for prognosis in aSAH patients.

## Data Availability

The data that support the findings of this study are available from the corresponding author upon reasonable request.
